# Short-Term Calorie Restriction Maintains Plasma Insulin Concentrations along with a Reduction in Hepatic Insulin-Degrading Enzyme Levels in db/db Mice

**DOI:** 10.3390/nu13041190

**Published:** 2021-04-03

**Authors:** Yudai Nonaka, Reo Takeda, Yutaka Kano, Daisuke Hoshino

**Affiliations:** 1 Bioscience and Technology Program, Department of Engineering Science, University of Electro-Communications, 1-5-1 Chofugaoka, Chofu, Tokyo 182-8585, Japan; takeda@ecc.pc.uec.ac.jp (R.T.); kano@pc.uec.ac.jp (Y.K.); dhoshino@uec.ac.jp (D.H.); 2 Japan Society for the Promotion of Science (JSPS), Kojimachi, Chiyoda-ku, Tokyo 102-0083, Japan

**Keywords:** calorie restriction, glucose transporter 4 (GLUT-4), insulin-degrading enzyme (IDE), diabetes

## Abstract

Maintaining blood insulin levels is important for patients with diabetes because insulin secretion capacity declines with the development of the disease. Calorie restriction (CR) is effective for the improvement of glucose tolerance, but it is not clear whether CR can maintain insulin levels in the late stage of diabetes. We examined the effect of CR on whole-body glucose tolerance and fasting blood insulin concentrations in the late stage of diabetes. Male db/db mice were subjected to either a standard laboratory diet ad libitum for 3 weeks (dbdb group) or 40% CR (dbdb+CR group). CR significantly decreased body mass and epididymal fat weight. Glucose tolerance and fasting glucose levels were significantly improved with 3-week CR. Fasting insulin concentrations were decreased in the dbdb group but were maintained in the dbdb+CR group. CR significantly reduced insulin-degrading enzyme (IDE) levels in the liver, and hepatic IDE levels were significantly positively and negatively correlated with plasma glucose concentrations (area under the curve) after glucose administration and after fasting insulin concentrations, respectively. Therefore, 3-week CR maintained blood insulin levels and improved glucose tolerance with decreased hepatic IDE levels in an animal model of late-stage diabetes.

## 1. Introduction

Type 2 diabetes mellitus (T2DM) increases the risk of hypertension and cardiovascular disease. The global epidemic of T2DM is a major public health problem. Disease progression is known to be caused by excessive fat accumulation. In fact, 60–90% of all patients with T2DM are obese (body mass index (BMI) ≥ 30 kg/m^2^)) or overweight (BMI ≥ 25 kg/m^2^) [1]. The global epidemic of obesity can explain the dramatic elevation in the incidence and prevalence of T2DM. Excessive fat accumulation induces insulin resistance in peripheral tissue [2] and results in glucose intolerance [3,4]. In contrast, weight loss by gastric bypass decreases BMI, which is used as an indicator of obesity, and increases the whole-body glucose disposal rate [5]. Therefore, a decrease in fat accumulation can prevent and reverse the development of T2DM.

Calorie restriction (CR) is an efficacious dietary intervention to abrogate the accumulation of visceral fat. In fact, mild and/or moderate weight loss with a moderate decline in calorie intake is recommended for patients with T2DM [6]. It has been reported that ~40% CR reduces body fat volume and fasting blood glucose and insulin levels and improves whole-body glucose tolerance in human [7,8] and animal experiments [9,10,11]. Fat reduction is associated with the CR-induced improvement of metabolism, but glucose uptake in peripheral tissue is also enhanced with CR. For example, CR leads to enhanced glucose transport with an increase in the levels of glucose transporter 4 (GLUT-4), which is a major transporter of glucose in skeletal muscle cells and in the cell surface membranes of insulin-stimulated muscle [12,13]. In addition, GLUT-4 levels in the adipose tissue have been reported to be important for maintaining whole-body glucose homeostasis [14,15,16]. Therefore, CR is an effective dietary intervention to prevent and ameliorate glucose intolerance due to its reduction in fat accumulation and improvement of glucose disposal and uptake in skeletal muscles and adipose tissues. The effect of CR on peripheral tissue has been studied, but the effect of CR on insulin levels, rather than on insulin action, has not been completely elucidated.

The pathophysiology of T2DM involves not only insulin resistance in peripheral tissue but also the impairment of insulin secretion capacity, particularly in the late stage of diabetes. As a consequence of long-term insulin resistance, the reduction in the number of functional β cells leads to a deficiency in insulin secretion [17,18,19]. Insulin secretory capacity declines gradually with the progression of T2DM in human patients [17,20]. Most T2DM patients eventually require insulin therapy. Therefore, in the late stage of the disease, the maintenance of blood insulin levels is crucial in terms of therapy. As CR improves insulin secretion capacity in rats with obesity induced by intake of a high-fat diet [21], it is possible that CR prevents the reduction of blood insulin concentrations. 

In the regulation of blood insulin levels, insulin-degrading enzyme (IDE) and carcinoembryonic antigen-related cell adhesion molecule 1 (CEACAM1), which promotes receptor-mediated insulin endocytosis and degradation in liver, also play a critical role [22]. IDE expression is altered by various nutritional and physiological factors, such as exercise and dietary intervention [23,24]. Therefore, we hypothesized that CR maintains blood insulin levels in the late stage of diabetes with an elevation in insulin secretion and/or a reduction in IDE expression. To examine this hypothesis, we conducted this study to examine the effect of CR on whole-body glucose tolerance and blood insulin levels with its related proteins in the late stage of diabetes. We used mice with a mutation in the leptin receptor gene (db/db), which leads to insulin resistance in the early stage of T2DM, and deficiency of insulin secretion in the late stage of diabetes [25].

## 2. Materials and Methods

### 2.1. Animal Treatment

Nine-week-old male db/db mice were housed individually under a 12:12-h light:dark cycle in a temperature-controlled room (23 °C). Mice were given a standard laboratory diet (CE-2; CLEA Japan, Tokyo, Japan) and water ad libitum. The mice were acclimated to the housing facility for 1 week. To examine the effects of CR on late-stage diabetes, we started CR intervention from 10 weeks old, as the previous study showed a decrease in blood insulin concentrations in db/db mice at 10–12 weeks [25].

After the acclimation period, the mice were divided into two groups that matched their body weights, blood glucose concentrations, and food intake during the acclimation period: one group continued to receive the standard diet ad libitum for the entire 21-day experimental period (dbdb group; *n* = 5), and the second group received the standard diet equal to 60% of the average amount of food eaten by the dbdb group during the 21-day experimental period to decrease their body weight (dbdb+CR group; *n* = 5). Misty mice of the same age were obtained from Japan SLC and treated for the same period (*n* = 5). All mice were allowed to drink water freely during the 21-day dietary intervention. Body weight and the amount of food intake were recorded daily throughout the dietary intervention, and fasting plasma glucose levels were measured weekly after 4-h fasting (morning fasting, 06:00–10:00). The University of Electro-Communications Institutional Animal Care and Use Committee approved all animal experiments in this study (No. 31).

### 2.2. Oral Glucose Tolerance Test

The day before the oral glucose tolerance test (OGTT), the dbdb and dbdb+CR groups had access to food ad libitum until 22:00 (food was removed after 22:00). After fasting for 12 h, oral administration of glucose (1 g/kg body weight) was performed using a gavage needle with a ball tip. Blood was drawn from the tail vein and harvested in micro-hematocrit capillary tubes containing heparin (Thermo Fisher Scientific, Waltham, MA, USA) before and at 10, 30, 60, and 120 min after the glucose administration. After the samples were centrifuged at 10,000 rpm for 5 min, the samples of plasma in the tubes were stored at −80 °C until analysis.

### 2.3. Tissue Collection

At the end of the experimental intervention, the mice were sacrificed under anesthesia using isoflurane without fasting. The tibial anterior (TA) muscle and liver were harvested and weighed. Then, the epididymal fat was harvested and weighed. We focused on epididymal fat because the weights are positively correlated with glucose concentrations during glucose tolerance test [26]. Liver, muscle, and fat samples were frozen at −80 °C until analysis.

Mice fasted for 4 h (morning fasting) before the blood glucose and insulin measurements at 10 and 13 weeks. Blood samples were drawn from the tail vein and harvested into micro-hematocrit capillary tubes covered with heparin (Thermo Fisher Scientific, Waltham, MA, USA). Plasma samples were separated by centrifugation at 10,000 rpm for 5 min and were stored at −80 °C until analysis.

### 2.4. Plasma Glucose and Insulin Concentrations

Concentrations of plasma glucose and insulin were measured using the Glucose C2 Test Wako Kit (Fujifilm Wako Pure Chemical Co., Osaka, Japan) and the Mouse Insulin ELISA Kit (Mercodia AB, Uppsala, Sweden), respectively.

### 2.5. Tissue Homogenization

Frozen TA muscle, epididymal fat, and liver were homogenized in an ice-cold RIPA lysis buffer (EMD Millipore, Temecula, CA) containing 0.25% deoxycholic acid, 50 mM Tris-HCl, 150 mM NaCl, 1% NP-40, 1 mM EDTA (pH 7.4), phosphatase inhibitors (PhosSTOP; Roche, Basel, Switzerland), and a protease inhibitor cocktail (Sigma-Aldrich, St. Louis, MO, USA). The homogenates were subjected to 3 freeze–thaw cycles to destroy intracellular organelles and rotated continuously at 4 °C for 60 min to solubilize the protein. The homogenized tissue samples were centrifuged at 700× *g* for 5 min at 4 °C, and the supernatants were harvested. Total protein content per tissue was quantified with a BCA Protein Assay Kit (Pierce, Rockford, IL, USA).

### 2.6. Western Blotting

The samples were mixed with sample buffer (Thermo Fisher Scientific, Waltham, MA, USA) and heated for 5 min in a heating block at 95 °C. Sample protein was loaded onto gels in equal amounts, separated by sodium dodecyl sulfate–polyacrylamide gel electrophoresis (7.5% or 10% resolving gels), and transferred to polyvinylidene difluoride (PVDF) membranes at 200 mA for 90 min. The membranes were then blocked for 1 h at room temperature in Tris-buffered saline with 0.1% Tween 20 (TBS-T; 137 mM NaCl, 20 mM Tris base, pH 7.6) containing 5% (*w*/*v*) nonfat powdered milk for 5 min at room temperature in Bullet Blocking One solution (Nacalai Tesque, Kyoto, Japan). The membranes were incubated overnight at 4 °C with the specific primary antibody diluted 1:1000 in TBS-T containing 5% bovine serum albumin. The primary antibodies used were anti-IDE (sc-393887; Santa Cruz Biotechnology, Dallas, TX, USA), anti-CD66a (carcino-embryonic antigen-related cell adhesion molecule 1 (CEACAM1); 14-0661-80; Invitrogen, Carlsbad, CA, USA), and anti-GLUT-4 (ab33780; Abcam, Cambridge, UK). After incubation with the specific antibody, the membranes were incubated for 1 h at room temperature with secondary antibodies (anti-mouse IgG, NA931 or anti-rabbit IgG, NA9340; Cytiva, Marlborough, MA, USA) and diluted 1:5000 in TBS-T containing 1% nonfat powdered milk. Bands were visualized by the Chemi-Lumi One Reagent (Nacalai Tesque, Kyoto, Japan) and quantified by Image Studio (LI-COR, Lincoln, NE, USA). Ponceau staining was performed to verify equal loading of samples.

### 2.7. Glucose-Stimulated Insulin Secretion of Isolated Islets

Another set of db/db mice (each group *n* = 5) was subjected to CR as described above, their islets were isolated, and glucose-stimulated insulin secretion was measured. As described previously [27], the islets were isolated by a method for collagenase digestion of the pancreas from euthanized mice. The mice were sacrificed after the dietary intervention, and the common bile duct was cannulated using a needle (Natsume Seisakusho, Tokyo, Japan). Then, Dulbecco’s modified Eagle’s medium (2–3 mL) containing 2 mg/mL collagenase IV (GIBCO, Carlsbad, CA, USA) was injected into the pancreas, and the pancreas was removed from the surrounding tissues. The removed pancreas was incubated in the solution for 35 min at 37 °C for tissue digestion. The digested tissue was washed twice with RPMI-1640 medium. Five size-matched islets were incubated for 30 min in 300 uL Krebs-Ringer buffer (15 mM HEPES pH 7.4, 2 mM CaCl_2_, 5 mM KCl, 1 mM MgCl_2_, 120 mM NaCl, 24 mM NaHCO_3_, 0.1% bovine serum albumin, and 2.8 mM glucose). After the incubation, the isolated islets were stimulated with high or low glucose, during which the islets were incubated for 60 min with 16.7 mM high-glucose Krebs-Ringer buffer or 2.8 mM low-glucose Krebs-Ringer buffer, respectively. The insulin concentration in the buffer containing the islets was measured using a Mouse Insulin ELISA Kit (Mercodia AB, Uppsala, Sweden).

### 2.8. Statistical Analysis

Data are represented as the mean ± standard error of the mean (SEM). For the data of the OGTT, a two-way analysis of variance (Prism ver.8 Software; GraphPad, San Diego, CA, USA) was used to test the effects of time and CR in db/db mice. For the other experiments, Student’s *t*-test was used to test statistical differences between the values obtained from the dbdb and dbdb+CR groups. Pearson’s correlation coefficient was used for correlation analysis. Statistical significance was set at a *p*-value of <0.05.

## 3. Results

### 3.1. Body Weight, Epididymal Fat Weight, and Total Food Intake

**Figure 1:**
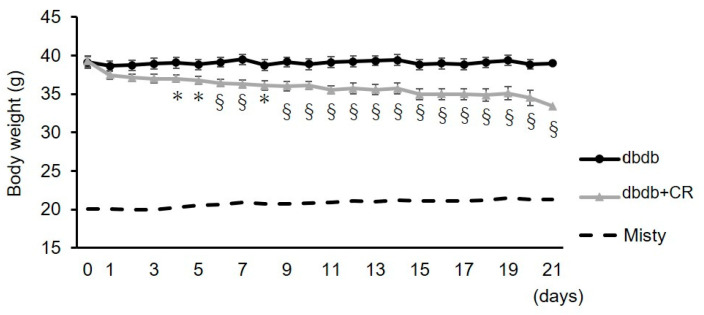
Changes in the body weight of rats during a 21-day dietary intervention. dbdb, ad libitum-fed control group; dbdb+CR, daily calorie restriction group. Data of misty mice were used as reference values (dotted lines). Data are the mean ± SEM (*n* = 5). * *p* < 0.05, ^§^ *p* < 0.01 compared with the dbdb group.

Changes in body weight during the 3-week dietary intervention are shown in Figure 1. During the intervention period, daily CR in the dbdb+CR group for 3 weeks induced a substantial decrease in body weight. The final body weight was approximately 14% lower in the dbdb+CR group than in the dbdb group (*p* < 0.01; Table 1). Total food intake during the 3-week experimental period was ~40% lower in the dbdb+CR group than in the dbdb group (*p* < 0.001; Table 1). Liver weight and epididymal fat weight were also lower in the dbdb+CR group than in the dbdb group (liver weight: *p* < 0.01; epididymal fat weight: *p* < 0.05; Table 1).

### 3.2. Fasting Plasma Glucose and Insulin Concentrations

At the completion of the 21-day dietary intervention, fasting plasma glucose concentrations were lower in the dbdb+CR group than in the dbdb group (*p* < 0.05; Table 1). Although there was no significant difference in fasting plasma insulin concentrations between both groups (Table 1), there was a significant difference in the change in fasting blood glucose and insulin concentrations between the two groups (both *p* < 0.05; Table 1).

### 3.3. OGTT

**Figure 2:**
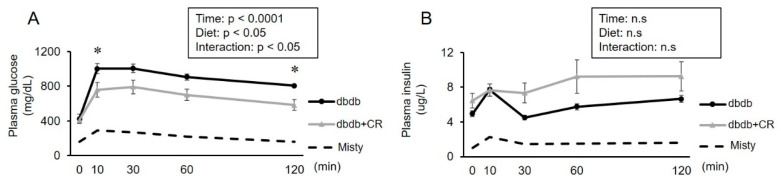
Effects of calorie restriction on glucose tolerance in db/db mice. Plasma glucose (**A**) and insulin (**B**) concentrations after oral glucose administration. dbdb, db/db ad libitum-fed control group; dbdb+CR, daily calorie restriction group. Data of misty mice were used as reference values (dotted lines). Data are the mean ± SEM (*n* = 5). * *p* < 0.05 compared with the dbdb group.

To test the effects of CR on whole-body glucose tolerance, we conducted an OGTT. The mice in the dbdb group became more hyperglycemic than those of the dbdb+CR group in response to glucose administration (Figure 2). The elevation in plasma glucose concentrations was lower in the dbdb+CR group than in the dbdb group at 10 and 120 min after glucose injection (10 min: *p* < 0.05; 120 min: *p* < 0.05; Figure 2). Plasma insulin concentrations did not significantly differ between the two groups.

### 3.4. GLUT-4 Protein Levels in TA Muscle and Epididymal Adipose Tissue

**Figure 3:**
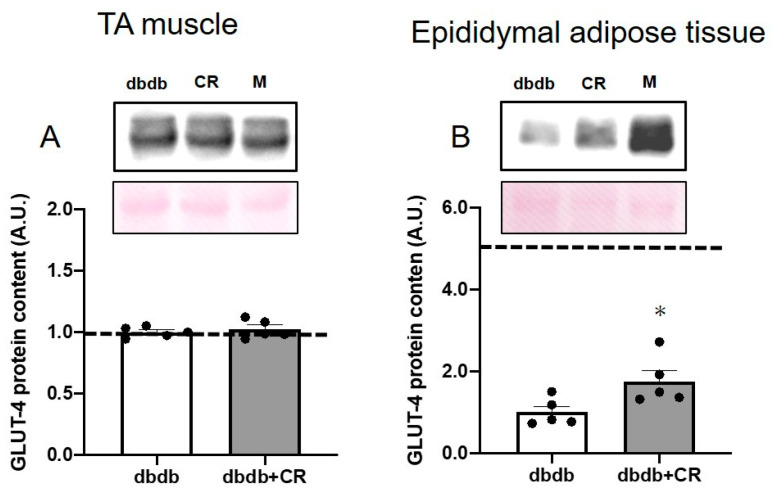
Effects of calorie restriction on GLUT-4 levels in db/db mice. GLUT-4 protein levels in mouse TA (tibialis anterior) muscle (**A**) and epididymal adipose tissue (**B**). dbdb, ad libitum-fed control group; dbdb+CR, daily calorie restriction group. Data of misty mice were used as reference values (dotted lines). Data are the mean ± SEM (*n* = 5). * *p* < 0.05 compared with the dbdb group.

GLUT-4 protein levels in epididymal adipose tissue, but not TA muscle, were significantly higher in the dbdb+CR group than in the dbdb group (*p* < 0.05; Figure 3).

### 3.5. Glucose-Stimulated Insulin Secretion in Isolated Islets

**Figure 4:**
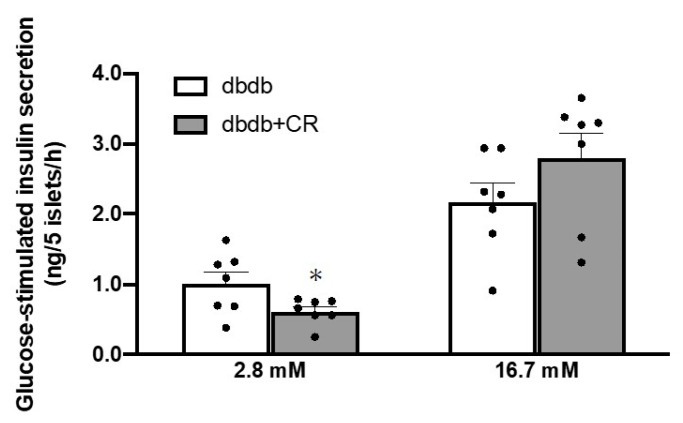
Effects of calorie restriction on glucose-stimulated insulin secretion in db/db mice. dbdb, ad libitum-fed control group; dbdb+CR, daily calorie restriction group. Data of misty mice were used as reference values (dotted lines). Data are the mean ± SEM (*n* = 7). * *p* < 0.05 compared with the dbdb group.

After the 3-week intervention period, pancreatic islets isolated from the mice in both groups were used for the measurement of glucose-stimulated insulin secretion. Insulin secretion in response to low-glucose treatment (baseline, 2.8 mM glucose) was lower in the dbdb+CR group than in the dbdb group (*p* < 0.05; Figure 4). Insulin secretion in response to high-glucose stimulation (16.7 mM glucose) did not differ between groups. 

### 3.6. IDE Protein Levels in TA Muscle and Liver and CEACAM1 Protein Levels in Liver

**Figure 5:**
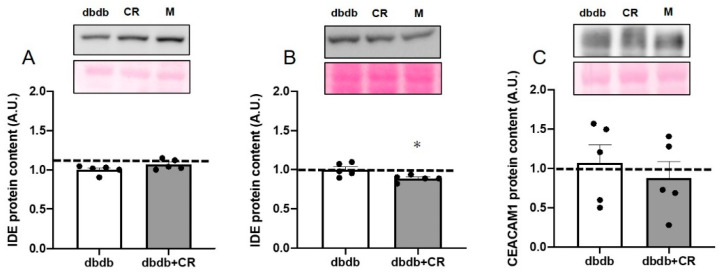
Effects of calorie restriction on insulin-degrading enzyme (IDE) and carcino-embryonic antigen-related cell adhesion molecule 1 (CEACAM1) levels in db/db mice. IDE protein levels in mouse tibialis anterior muscle (**A**) and liver (**B**) and CEACAM1 protein levels in liver (**C**). dbdb, ad libitum-fed control group; dbdb+CR, daily calorie restriction group. Data of misty mice were used as reference values (dotted lines). Data are the mean ± SEM (*n* = 5). * *p* < 0.05 compared with the dbdb group.

**Figure 6:**
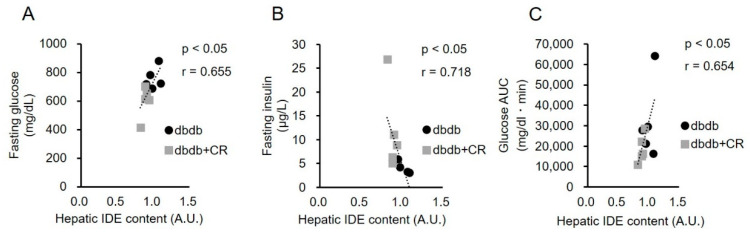
Correlations between hepatic insulin-degrading enzyme (IDE) levels and fasting glucose (**A**) or fasting insulin (**B**) concentrations or glucose area under the curve (AUC) during the oral glucose tolerance test (OGTT) (**C**). dbdb, ad libitum-fed control group; dbdb+CR, daily calorie restriction group.

We explored the mechanism by which fasting insulin concentrations were maintained with the CR intervention. While there were no significant differences in IDE protein expression in the TA muscle and CEACAM1 levels in the liver between the two groups, the hepatic IDE protein levels were lower in the dbdb+CR group than in the dbdb group (*p* < 0.05; Figure 5). Hepatic IDE protein levels were positively correlated with fasting glucose concentrations (r = 0.655, *p* < 0.05; Figure 6). Hepatic IDE levels were negatively correlated with concentrations of plasma insulin (r = 0.718, *p* < 0.05; Figure 6). In addition, hepatic IDE protein levels were significantly correlated with the plasma glucose area under the curve (AUC) during the OGTT (r = 0.654, *p* < 0.05; Figure 6).

## 4. Discussion

Our study was conducted to examine the effects of 3-week CR on glucose tolerance and fasting insulin concentrations in the late stage of diabetes. We discovered that 3-week CR in db/db mice improved glucose tolerance, with a decline in epididymal adipose tissue and an increase in fat GLUT-4 levels, and maintained fasting insulin concentrations, accompanied by decreased IDE levels in the liver.

Three-week CR improved glucose tolerance, such as lowered plasma glucose concentrations during the OGTT (Figure 2A). The CR-induced improvement of glucose tolerance in db/db mice was consistent with the results of previous studies that investigated the effects of CR on glycemic improvement and glucose metabolism [10,11,21]. As insulin concentrations during the OGTT did not differ between the two groups (Figure 2A), we estimated that the reason for the improvement in the OGTT was also the reason for the increase in glucose uptake in peripheral tissue. However, there was no significant change in GLUT-4 levels, a major glucose transporter in skeletal muscle cells [28], with 3-week CR (Figure 3A). We did not measure the parameters of insulin sensitivity of skeletal muscle, but previous studies reported that CR increased GLUT-4 levels in the cell surface membranes of insulin-stimulated muscle [12] without a change in total GLUT-4 protein expression [13]. It is likely that insulin sensitivity was improved by 3-week CR without changes in total GLUT-4 levels. The GLUT-4 protein in adipose tissue is also associated with whole-body insulin resistance [15,29]. Interestingly, CR significantly up-regulated GLUT-4 protein levels in adipose tissue (Figure 3B). This increase in GLUT-4 content in epididymal adipose tissue would contribute, in part, to the improvement of whole-body glucose tolerance, because previous studies reported that diabetes decreases adipose GLUT-4 expression and impairs glucose tolerance [29] and revealed that quantitative alterations of adipose GLUT-4 levels alter whole-body glucose tolerance, such as skeletal muscle insulin resistance in transgenic mice [14,15,30]. Furthermore, since adipose tissue accounts for ~10% of the insulin-stimulated whole-body glucose uptake [16], an increase in GLUT-4 protein levels after the CR intervention would contribute, in part, to the improvement of insulin-stimulated glucose uptake into the adipose tissue. In addition, CR induced an approximately 10% reduction in epidydimal fat weight in the present study (Table 1). Since excessive fat accumulation aggravates insulin sensitivity in peripheral tissue [3,4], the CR-induced reduction in adipose tissue weight may also be implicated in the improvement of insulin sensitivity in skeletal muscle and adipose tissue. Therefore, these data suggest that the CR-induced improvement of glucose tolerance during the OGTT was associated with an increase in adipose GLUT-4 levels and a reduction in epididymal adipose tissue weight.

Fasting insulin concentrations decreased from 10 to 13 weeks of age in the dbdb group, but they were maintained in the dbdb+CR group (Table 1), indicating that CR can inhibit the reduction of fasting blood insulin concentrations shown in the late stage of diabetes. To clarify whether this maintenance of fasting plasma insulin concentrations was due to alternations in insulin secretion and/or degradation, we measured insulin secretion rates in extracted islets and the levels of IDE protein. Insulin secretion in response to low-glucose stimulation, but not high-glucose, was significantly lower in the dbdb+CR group than in the dbdb group (Figure 4). This result is comparable with the results of a previous study in which insulin secretion in response to low-glucose stimulation (2.8 mM) was reduced after 3-week CR [21]. Therefore, we consider that insulin degradation, rather than insulin secretion, may have played a role in the maintenance of fasting blood insulin concentrations in the CR group in this study. In fact, 3-week CR significantly decreased IDE expression levels in the liver but did not change hepatic CEACAM1 or muscle IDE expression levels (Figure 5). Furthermore, there was a significant negative correlation between IDE expression in the liver and fasting insulin concentrations in the blood (Figure 6B). This relationship suggests that the reduction in IDE expression contributes to the inhibition of insulin deficiency in the late stage of diabetes. This is supported by previous studies in which short-term IDE knockout can increase fasting insulin levels [31,32]. Taken together, these results suggest that short-term CR in the late stage of diabetes, with decreases in blood insulin levels, can help to maintain blood insulin levels, potentially by reducing IDE levels.

Insulin sensitivity in peripheral tissue and insulin secretion from β cells in the pancreas are considered important for glucose disposal [33]. Our study provides the novel finding that a decrease in IDE can improve glucose disposal in the late stage of diabetes. This is because a significant positive correlation was shown between IDE levels in the liver and fasting glucose concentrations or glucose AUC during an OGTT (Figure 6C). However, previous studies reported that IDE levels had a positive effect on glucose tolerance [34,35]. For example, whole-body and liver-specific knockout of IDE impairs fasting glucose levels and glucose tolerance [32,36,37]. An exercise-induced increase in IDE expression was shown to reduce glycemia and improve insulin sensitivity in mice [24]. We consider that the differences in the results between previous studies and ours are because we used a late-stage model of diabetes, while previous studies used healthy animals. The resulting decrease in IDE levels in the present study may represent a specific effect of CR in late-stage diabetes. In fact, there was no positive relationship between IDE levels and fasting glucose concentrations or glucose AUC during the OGTT when correlations were analyzed in this study, including for the healthy controls. A previous study also found that the effects of a dipeptidyl peptidase-IV inhibitor on plasma insulin concentrations were different between late- and early-stage models of diabetes [38]. In end-stage diabetes, in which blood insulin concentrations are lowered, the adaptation to CR or other pharmacological and nutritional interventions is partially different from that observed in healthy subjects. Therefore, as the practical significance of this study, the CR-induced reduction in IDE expression would be a useful therapeutic intervention for patients in the late stage of diabetes. 

This study has several limitations. It is not clear whether insulin concentration is maintained in late-stage human patients when their hepatic IDE protein levels are decreased. Furthermore, we need to examine sex differences in effects of CR in late-stage of the disease because we used only male mice and there are differences between male and female mice in health and longevity benefits with CR [39]. In this study, Mice performed 12 h fasting and 4 h fasting before the measurements of blood glucose and insulin concentrations. Fasting itself may induce CR effects but they would be little because 3-day CR (40% CR) did not alter glucose tolerance but 10-day CR induced improvement of glucose tolerance [10]. We need to clarify these issues in the future study. 

In conclusion, short-term CR in an animal model of late-stage diabetes increased adipose tissue GLUT-4 levels and reduced epididymal adipose tissue weight, leading to an improvement in whole-body glucose tolerance. The CR-induced improvement in blood glucose concentrations was associated with the maintenance of fasting blood insulin levels and decreased IDE expression in the liver.

## Figures and Tables

**Table 1:** Body weight, total food intake, liver weight, epididymal fat weight, and concentrations of plasma glucose and insulin in mice.

	dbdb	dbdb+CR	Misty
Initial body weight (g)	39.1 ± 0.7	39.2 ± 0.7	20.0 ± 0.3
Final body weight (g)	39.0 ± 0.7	33.4 ± 0.9 **	21.2 ± 0.2
Total food intake (g)	107 ± 4	64 ± 1***	72 ± 1
Liver weight (g)	2.1 ± 0.1	1.9 ± 0.1 **	1.3 ± 0.1
Epidydimal fat weight (g)	1.7 ± 0.1	1.5 ± 0.1 *	0.3 ± 0.1
Plasma glucose concentration (10 weeks) (mg/dL)	672 ± 18	644 ± 67	167 ± 7
Plasma glucose concentration (13 weeks) (mg/dL)	759 ± 34	599 ± 24 *	199 ± 6
ΔPlasma glucose concentration (13 vs.10 weeks) (mg/dL)	87 ± 28	−45 ± 36 *	32 ± 7
Plasma insulin concentration (10 weeks) (µg/L)	8.2 ± 1.7	9.5 ± 2.5	0.5 ± 0.1
Plasma insulin concentration (13 weeks) (µg/L)	4.4 ± 0.6	11.6 ± 4.0	0.3 ± 0.1
ΔPlasma insulin concentration (13 vs. 10 weeks) (µg/L)	−3.7 ± 1.8	2.1 ± 1.7 *	−0.2 ± 0.1

Data are the mean ± SEM, *n* = 5. * *p* < 0.05, ** *p* < 0.01, and *** *p* < 0.001, compared with the dbdb group. The mice fasted for 4 h for measurement of blood glucose and insulin concentration. dbdb, ad libitum-fed control group; dbdb+CR, daily calorie restriction group.

## Data Availability

The data presented in this study are available on request from the corresponding author.
